# The role of the microcirculation in muscle function and plasticity

**DOI:** 10.1007/s10974-019-09520-2

**Published:** 2019-06-05

**Authors:** Paul Hendrickse, Hans Degens

**Affiliations:** 1 Research Centre for Musculoskeletal Science & Sports Medicine, School of Healthcare Science, Manchester Metropolitan University, John Dalton Building; Chester Street, Manchester, M1 5GD UK; 2 Lithuanian Sports University, Kaunas, Lithuania; 3 University of Medicine and Pharmacy of Targu Mures, Targu Mures, Romania

**Keywords:** Capillary, Muscle, Hypertrophy, Oxidative capacity, Angiogenesis, Microcirculation

## Abstract

It is widely acknowledged that maintenance of muscle, size, strength and endurance is necessary for quality of life and the role that skeletal muscle microcirculation plays in muscle health is becoming increasingly clear. Here we discuss the role that skeletal muscle microcirculation plays in muscle function and plasticity. Besides the density of the capillary network, also the distribution of capillaries is crucial for adequate muscle oxygenation. While capillaries are important for oxygen delivery, the capillary supply to a fibre is related to fibre size rather than oxidative capacity. This link between fibre size and capillary supply is also reflected by the similar time course of hypertrophy and angiogenesis, and the cross-talk between capillaries and satellite cells. A dense vascular network may in fact be more important for a swift repair of muscle damage than the abundance of satellite cells and a lower capillary density may also attenuate the hypertrophic response. Capillary rarefaction does not only occur during ageing, but also during conditions as chronic heart failure, where endothelial apoptosis has been reported to precede muscle atrophy. It has been suggested that capillary rarefaction precedes sarcopenia. If so, stimulation of angiogenesis by for instance endurance training before a hypertrophic stimulus may enhance the hypertrophic response. The microcirculation may thus well be a little-explored target to improve muscle function and the success of rehabilitation programmes during ageing and chronic diseases.

## Introduction

Skeletal muscle comprises almost 40% of the human body. They contain a dense capillary network that serves to deliver oxygen and nutrients, and remove waste products and heat from the skeletal muscle cells. This becomes particularly important during exercise, where the metabolic rate can increase 30-50-fold (Payne and Bearden 2006) with a commensurate increase in oxygen demand that is realised by increased blood flow and capillary recruitment characterised by elevated red blood cell flux and velocity (Poole 2004) as seen already now 100 years ago by Krogh (1919). The increased red blood cell (RBC) flux and velocity enhances oxygen diffusion from the capillary to the mitochondria in the muscle fibres (Poole 2004). Krogh also noted that in skeletal muscle the capillaries largely run parallel to the muscle fibres, an orientation that helped the development of his model of tissue oxygenation (Krogh 1919). While this is the case at longer sarcomere lengths, at shorter sarcomere length, the capillaries become more tortuous, resulting in a larger contact area between the capillaries and the muscle fibres that facilitates oxygen diffusion (Poole 2004). Capillaries are arranged in microvascular units, defined by the terminal arteriole and the 10–20 capillaries it feeds, and it is in the arterioles that the perfusion of the downstream capillaries is regulated via vasomotion (Wagenmakers et al. 2016). The capillaries drain into collecting venules that merge into collecting veins (Hudlicka 2011; Korthuis 2011).

### Blood flow

The increased metabolic rate during muscle contraction is accompanied by an up to 100-fold rise in blood flow (Poole et al. 2011). A contracting muscle is, however, in a paradoxical situation as with increasing force not only the oxygen demand rises, but also the intramuscular pressure, and even more so during shortening contractions (Degens et al. 1998), that will impede blood flow. The rise in intramuscular pressure, irrespective of the size of the muscle, can even cause a complete cessation of blood flow and result in the deoxygenation during sustained isometric contractions as low as 30% maximal force (de Ruiter et al. 2007). While during intermittent contractions the blood flow is enhanced during the relaxation phase, it has been reported that the cessation of flow during the contraction phase is such that increasing the duty cycle beyond 10% does not result in a further increase in blood flow (Degens et al. 1998).

## Capillarisation in skeletal muscle

### Capillary density and muscle oxidative capacity

Given the importance of the capillary bed for the delivery of oxygen to the muscle, it is expected that highly oxidative muscles have a denser capillary network than highly glycolytic muscles. Particularly in rodents, there is a large difference in the fibre type composition of skeletal muscle, where for instance in the soleus muscle 87% and in the extensor digitorum longus (EDL) muscle only 2% of the fibres are type I (S) fibres (Armstrong and Phelps 1984). The soleus muscle also has a higher oxidative capacity and capillary density (CD: the number of capillaries mm^−2^ of muscle) than the EDL (Gray and Renkin 1978; Egginton et al. 1988). Such differences in CD are even seen within a muscle, where for instance in the rat plantaris muscle the deep oxidative region has a higher CD than the more glycolytic superficial region of the muscle (Wust et al. 2009; Hudlicka 2011). The link between oxidative metabolism and the density of the capillary network in the muscle is also reflected between muscles from different species. For instance, in the highly active flight muscles of the hummingbird with maximal respiration rates more than twice that found in mammals, capillaries make up a 2–6 times greater proportion of the muscle volume than that seen in mammalian hind limb muscles (Suarez et al. 1991). In fact, it has been suggested that in the humming bird flight muscles, the mitochondrial volume density and inner membrane density are near their theoretical upper limit to maximise respiratory capacity (Suarez et al. 1991).

The capillary supply to a muscle is highly adaptable to altered functional demands. One such adaptation is the increased CD in the flight muscle of pigeons after 2 months of cold exposure concomitant with an increase in mitochondrial density to meet the metabolic demands of shivering (Mathieu-Costello et al. 1998). Likewise, endurance athletes have a higher oxidative capacity and CD than sedentary people (Tesch et al. 1984; Saltin et al. 1977). Weightlifters and powerlifters, on the other hand, participate in activities that require a few maximal contractions that do not require the aerobic generation of ATP and therefore have a lower demand for oxygen delivery by the circulatory system. Accordingly, they have a lower CD than sedentary people and endurance athletes (Tesch et al. 1984). All these observations suggest that an important function of the capillary bed is the delivery of oxygen to the working mitochondria.

### Distribution of capillaries

**Fig. 1:**
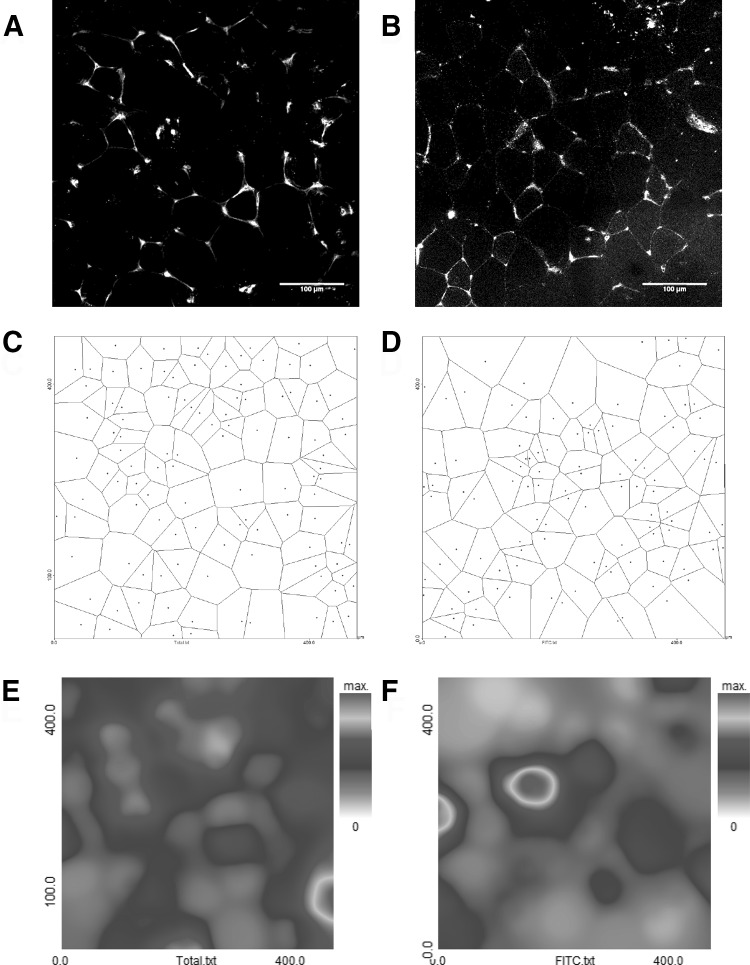
**a**, **b** Images of rat skeletal muscle stained for capillaries with fluorescently-labelled lectin. Both images have the same capillary density but a different heterogeneity of capillary spacing. In image **a** the logarithmic standard deviation of the domain radii (log_R_SD) is 0.093 and in **b** it is 0.121. Capillary domains (estimates of oxygen supply areas) for **a**, **b** are shown in **c**, **d**, respectively. **e**, **f** are heat maps for capillary distribution and give a rough indication of the distribution of oxygen partial pressure in the tissue. Unpublished observations

Most studies on muscle capillarisation report the CD and the capillary to fibre ratio (C:F). While these measures give a good reflection of the size of the capillary network in the muscle, they give no information of the distribution of the capillaries. Model calculations have shown that an increased heterogeneity of capillary spacing has a negative impact on muscle oxygenation (Piiper and Scheid 1991; Turek et al. 1992; Degens et al. 2006). Given the significance, it is somewhat surprising that the distribution of capillaries has received little attention. This may be partly attributable to the lack of techniques to obtain a measure of the heterogeneity of capillary spacing. The method of capillary domains (Fig. 1), where a capillary domain is defined as the area of tissue surrounding a capillary delineated from adjacent capillaries by equidistant boundaries, gives a quantitative measure of the heterogeneity of capillary spacing as the standard deviation of the logarithm of the domain areas (Hoofd et al. 1985). Using this method, it was found that the capillary distribution is more homogeneous in the oxidative soleus than the glycolytic EDL muscle, but no difference in slow and fast muscles of the eel was found, despite a 35-fold difference in CD (Egginton et al. 1988). It has been shown that the heterogeneity of capillary spacing correlates with the variation of fibre size (Degens et al. 2009; Barnouin et al. 2017), which might be explicable by the morphological constraints of positioning the capillaries on the surface of the fibre only.

With a theoretical model of tissue oxygenation it was found that in rat soleus muscle with a typical CD, working at the anaerobic threshold (2/3 of maximum oxygen consumption), the impact of changes in the heterogeneity of capillary spacing on tissue oxygenation was more pronounced than the effects of redistribution of flow (Hoofd and Degens 2009). Something similar was seen in heart muscle (Turek et al. 1992). An increased heterogeneity of the distribution of perfused capillaries has been seen in sepsis patients that in severe cases led, as predicted by the oxygenation models, to an impaired tissue oxygenation (Goldman et al. 2006; Walley 1996). These observations provide evidence that the distribution of capillaries in the muscle is (i) more important for a good tissue oxygenation than flow distribution and (ii) that capillaries are not randomly arranged, but that their positioning is controlled to ensure adequate muscle oxygenation.

## Capillarisation and muscle function

Evidence for the significance of the capillary network for muscle fatigue resistance comes from the observation that the increased fatigue resistance of chronically stimulated muscles was associated with an elevated CD (Lieber 1986; Pette and Vrbova 1992; Hudlicka et al. 1977). As the blood flow and oxidative capacity were also increased, it is difficult to disentangle to what extent the increased fatigue resistance in chronically stimulated muscles is attributable to an increase in oxidative capacity, blood flow and/or CD. However, the improved fatigue resistance in overloaded rat EDL (Egginton et al. 1998) and mouse plantaris muscles (Ballak et al. 2016) was accompanied by an increased CD, but without concomitant increases in blood flow or oxidative capacity, respectively. These observations indicate that the capillary network plays an important role in muscle fatigue resistance. This is further supported by an elegant study, in which a reduction in the number of functional capillaries through microsphere-induced arteriole occlusion resulted in a significant decline in performance of the isolated rat heart (Hauton et al. 2015).

## Impact on metabolism

Skeletal muscle is the major storage tissue for glucose (in the form of glycogen) that is delivered to the muscle via the capillary blood (Jensen et al. 2011). The elevated post-prandial circulating glucose concentration induces the release of insulin from the pancreas. The vasodilating effect of insulin in turn causes a modest increase in skeletal muscle blood flow that enhances the transport of glucose into the muscle tissue (Vincent et al. 2006; Wagenmakers et al. 2016). As the network responsible for the transfer of substances between the circulatory system and skeletal muscle, capillaries are crucial for muscle glucose uptake. In line with this, a low skeletal muscle CD was associated with glucose intolerance and insulin resistance in both young and old humans (Snijders et al. 2017b, Landers-Ramos and Prior 2018; Groen et al. 2014; Lillioja et al. 1987) and stroke patients (Prior et al. 2009). Moreover, an increased CD after training improved insulin sensitivity and glucose tolerance (Landers-Ramos and Prior 2018; Prior et al. 2015). The observation that acute occlusion of arterioles and downstream capillaries with 15-µm microspheres, without a change in blood flow, resulted in a significant reduction in insulin-mediated glucose uptake (Vollus et al. 2007) indicates that the capillary bed is perhaps more important for glucose tolerance than blood flow. Therefore, a diminished CD in disease or due to sedentary behaviour may thus contribute to the impaired insulin sensitivity and glucose intolerance in these conditions, which may be further aggravated by systemic inflammation and accumulation of fatty acid metabolites that both impair activation of the insulin signalling cascade (Wagenmakers et al. 2016).

## Size principle in relation to capillary supply to a fibre

### The size principle

The size principle is defined as the inverse relationship between the oxidative capacity and the cross-sectional area of a fibre that is explained by diffusion limitations (van der Laarse et al. 1998). This diffusion limitation would limit fibre size. There is, however, a metabolic advantage of having large fibres, where for instance the cost of Na^+^/K^+^-ATPase function was twice as large in muscles with small fibres, with a twofold higher surface to volume ratio, than that in muscles with large fibres (Jimenez et al. 2011). Such observations have led to the concept of an ‘optimal fibre size’, which suggests that in larger fibres there is a trade-off between ‘diffusion constraints’ and ‘metabolic cost savings’ (Johnston et al. 2006). In fish muscle, the diffusion constraint is overcome to some extent by intramyocyte lipid that increases the oxygen permeability of the tissue (Hoofd and Egginton 1997), or flattened fibres that increase the surface to volume ratio (Johnston 1982). Another adaptation is migration of mitochondria to the periphery of the fibre, as seen in the white fibres of sharks and rays (Kinsey et al. 2011). While advantageous in terms of shortening the diffusion distances for oxygen, it creates another problem, as the radial distance over which ATP has to diffuse from the mitochondria to the inner myofibrils is increased (Kinsey et al. 2011). The overcome this conundrum, the musculature of the blue crab displays a particularly interesting adaptation where their large aerobic fibres are subdivided into smaller well-perfused subdivisions to shorten the diffusion distances (Hardy et al. 2009).

In mammals, large muscle fibres are not subdivided, nor is there evidence of an increase in intramyocellular lipids and/or changes in fibre shape. Yet, there is evidence that also in mammals the inverse relationship between fibre size and oxidative capacity can be overcome. For example, oestrogen-related receptor gamma (Errγ) overexpression in myostatin null mice (which demonstrate a hypermuscular phenotype) resulted in an elevated oxidative capacity to similar levels to that found in fibres of wild type mice (Omairi et al. 2016). Also, in overloaded mouse plantaris muscle the oxidative capacity was elevated (Ballak et al. 2016) and in both cases this was accompanied with an increased CD. These data thus suggest that the alleged trade-off between muscle fibre size and oxidative capacity can also be broken in mammalian muscle, among others by capillary proliferation.

**Fig. 2:**
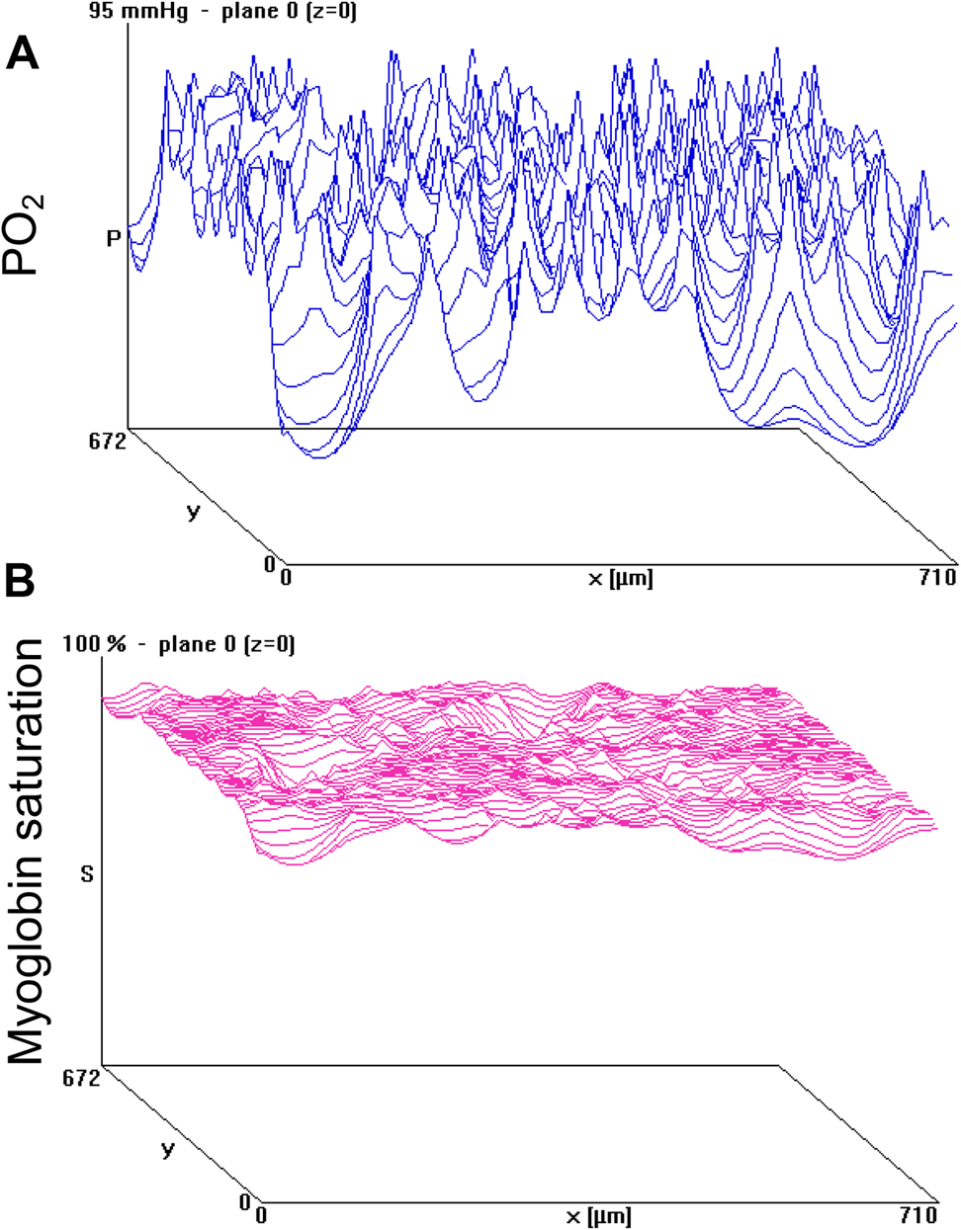
**a** Oxygen partial pressure (PO_2_ in mmHg) and **b** myoglobin saturation in a mouse soleus muscle working at maximal oxygen uptake, calculated using a mathematical model of tissue oxygenation (Hoofd 1995). Peaks in **a** illustrate capillary PO_2_ assumed to be 95 mmHg. Unpublished observations

At first glance, it seems that the violation of the size principle is made possible by a concomitant increase in capillary supply to a fibre, suggesting that the oxidative capacity of, and the capillary supply to, a fibre are linked. However, a study of human vastus lateralis and soleus muscle showed that the capillary supply to a fibre is not determined by its oxidative capacity, but rather by muscle fibre cross-sectional area (Bosutti et al. 2015). Together with the observation that long-term high frequency electrical stimulation of glycolytic fibres led to an increased capillary supply without an increase in oxidative capacity (Egginton and Hudlicka 2000), this suggests that oxidative capacity is not a determinant of capillary supply to a fibre and that capillarisation may play a more important role in metabolite removal than in substrate delivery. One explanation for the absence of a relationship between capillary supply to and oxidative capacity of a fibre is that even in the face of large variations in oxygen pressures in the tissue, the myoglobin saturation is, due to the shape of the myoglobin dissociation curve, rather homogeneous throughout the working muscle tissue (Fig. 2). This would ensure an adequate oxygen supply to the working mitochondria, even at a low oxygen partial pressure.

## Adaptations of the capillary network

### Muscle hypertrophy

Muscle has a remarkable ability to respond to altered functional demands. For instance, muscle hypertrophies in response to an overload stimulus, such as resistance exercise, and some models of compensatory hypertrophy even result in a doubling in muscle size (Degens 2012). For the development of such hypertrophy the delivery of nutrients, including amino acids, is essential. The amino acids and insulin facilitate hypertrophy via activation of mammalian target of rapamycin (mTOR) that stimulates protein synthesis through enhanced translation initiation and elongation (Fry and Rasmussen 2011; Timmerman et al. 2010). Besides stimulating protein synthesis, insulin also enhances nutritive flow to the muscle (Fry and Rasmussen 2011). Ultimately, the nutrients diffuse from the capillary blood into the muscle, and it is therefore likely that the density of the capillary network will have an impact on the development of hypertrophy. In addition, unlike in large fish muscle fibres, even at extreme levels of muscle hypertrophy mammalian skeletal muscle demonstrates a normal distribution of mitochondria (Chalmers et al. 1992). Without angiogenesis, the increase in fibre cross-sectional area pushes the capillaries apart, increasing the distance from the capillary to mitochondria in the interior of the fibre, thus putting a theoretical diffusion limitation on hypertrophy (Degens 2012).

The increase in diffusion distances during hypertrophy is attenuated by capillary proliferation in both rodent (Degens et al. 1992; Egginton et al. 1998) and human muscle (Verdijk et al. 2016). Also, during the more than eightfold increase in fibre size during maturational growth angiogenesis occurs (Ripoll et al. 1979; Degens et al. 2006). However, during both maturational and overload-induced muscle fibre growth the capillary proliferation is proportionally less than the increase in fibre size, resulting in a decreased CD (Ripoll et al. 1979; Hudlicka et al. 1992; Degens et al. 1992). Yet, the heterogeneity of capillary spacing is maintained, both during maturational growth (Degens et al. 2006) and overload-induced hypertrophy (Degens et al. 1992), and model calculations indicate that this is sufficient for adequate oxygenation (Degens et al. 2006). This indicates that capillary proliferation is not random, but such that muscle oxygenation is preserved.

The number of capillaries supplying a fibre is positively related to fibre size in both human (Ahmed et al. 1997; Bosutti et al. 2015) and rodent muscles (Degens et al. 1994; Wust et al. 2009). This suggests that fibre growth and capillary proliferation are coupled. Perhaps an even stronger indication of such a coupling is the observation that capillary proliferation and fibre growth follow a similar time course in both overloaded rodent muscles (Plyley et al. 1998) and in human muscles subjected to a resistance training stimulus (Verdijk et al. 2016; Green et al. 1999; Holloway et al. 2018).

Satellite cells (SCs) are thought to play an important role in muscle hypertrophy and regeneration (van der Meer et al. 2011). SCs are situated between the sarcolemma and basal lamina of a muscle fibre and act as a source of new myonuclei. Upon anabolic stimulation or muscle damage, SCs are activated and facilitate hypertrophy by fusing with an existing muscle fibre to donate a new myonucleus to maintain the nuclear domain size (van der Meer et al. 2011). Interestingly, 82 and 68% of SCs in mouse tibialis anterior and human deltoid muscle, respectively, are within 5 µm of a capillary, and capillaries and SCs can reciprocally activate each other, probably via diffusion of secreted growth factors (Christov et al. 2007). Indeed, in vitro endothelial cells stimulate SC growth through secretion of growth factors including IGF, HGF and VEGF, and in muscle tissue it was seen that active SCs were located closer to capillaries than quiescent SC (Christov et al. 2007). This suggests that their ability to differentiate may be related to their proximity to capillaries and hence diffusion limitations. Vice versa, microvascular fragments develop smooth muscle sprouts when co-cultured with SCs but not when cultured on their own (Rhoads et al. 2009). That such a relationship may play a significant role in vivo is suggested by the observation that greater capillarisation in the vastus lateralis was associated with an amplified activation/expansion SC response and accelerated repair after muscle damage caused by eccentric contractions (Nederveen et al. 2018). Additionally, greater activation of SCs in response to a 16-week resistance exercise is complemented with an increase in C:F (Nederveen et al. 2017), and the blunted hypertrophic response in old mouse muscle was not associated by a reduced number of SCs, but by an attenuated overload-induced increase in CD (Ballak et al. 2016). In addition, regeneration after cardiotoxin-induced muscle damage was better in mouse muscles with a larger C:F, even if they had a lower number of SCs (Omairi et al. 2016). In this context, it is interesting to note that the distance between SCs and capillaries is larger in muscles from older than young-adult humans (Nederveen et al. 2016) and it is possible that the impaired hypertrophic response and regenerative capacity in old age is due to diffusion limitations. These data indicate that there is significant cross-talk between capillaries and myosatellite cells. The coupling between fibre size and the capillary bed is, however, not limited to capillary—SC cross-talk, as also mature myofibres can release angiogenic growth factors, such as VEGF (Takahashi et al. 2002) and Follistatin-like I (Ouchi et al. 2008), that also stimulate muscle growth. In conclusion, there is significant evidence that capillaries play an important role in the hypertrophic response and muscle regeneration.

A denser capillary network not only resulted in a larger SC activation and expansion (Nederveen et al. 2018), but also a more pronounced hypertrophic response (Snijders et al. 2017a) to a resistance training programme. This may have implications for the design of rehabilitation programmes where stimulation of angiogenesis before starting a resistance-training programme could potentially improve the outcome. One way to explore this, is to precede resistance training with a period of endurance training that has been shown in both human (Gavin et al. 2015) and rat muscle to induce angiogenesis (Kurosaka et al. 2012). An alternative approach could be to employ concurrent training. While concurrent training has traditionally been associated with the “interference effect” (Hickson 1980) that was thought to compromise muscle hypertrophy, recent research suggests that careful selection of training modalities could prevent this phenomenon and even augment increases in fibre size (Murach and Bagley 2016).

As discussed above, there is a lose link between the capillary supply to a fibre and fibre size, and the time course of angiogenesis is similar to that of hypertrophy. Atrophy, however, is not associated with a concomitant proportional capillary rarefaction as reflected by the initial increase in CD after denervation (Paudyal et al. 2018; Degens et al. 2008). After longer periods of denervation further capillary loss occurs and even avascular regions may develop (Borisov et al. 2000). The early increase in CD may provide a window of opportunity for treatment of denervation-induced atrophy, where the elevated CD may be conducive for muscle growth and regeneration.

It thus appears that the relationship between capillary supply to and oxidative capacity of a fibre is maintained during muscle growth, whereas capillary rarefaction during atrophy is such that the CD is, at least transiently, elevated.

### Endurance exercise and chronic electrical stimulation

Prolonged endurance exercise training leads to an increase in CD. Based on observation in rodents, it has been suggested that the rate of angiogenesis is faster in response to large-volume low-intensity exercise than to low-volume high-intensity exercise (Olfert et al. 2016). Perhaps chronic low-frequency stimulation can be considered a proxy for large-volume low-intensity training and indeed does induce angiogenesis in as short a period as 2 days (Hudlicka et al. 1982) that may be due to increased sheer stress on the endothelial cells (Egginton et al. 2001; Olfert et al. 2016). Also in humans, training at both moderate and high intensities increases capillary supply to both type I and II fibres (Andersen and Henriksson 1977; Jensen et al. 2004; Gavin et al. 2015).

## Angiogenesis

Here we give a limited description of the process of angiogenesis and refer for further details to the review by Olfert et al. (2016). In short, the formation of new capillaries from existing blood vessels is known as angiogenesis and occurs in response to exercise and during wound repair. Two types of angiogenesis can be distinguished: splitting and sprouting (Olfert et al. 2016). Splitting angiogenesis, the longitudinal separation of capillaries, is thought to be a response to increased sheer stress to the lumen of capillaries due to hyperaemia (Olfert et al. 2016). Sprouting, the formation of capillaries from buds on pre-existing capillaries, occurs in response to stretch on the capillaries during conditions as overload (Zhou et al. 1998).

The increased blood flow during e.g. chronic electrical stimulation is attributable to vasodilation in response to nitric oxide (NO), prostacyclin, ATP and adenosine. The presence of NO in turn enhances expression of the vascular endothelial growth factor (VEGF), the most important angiogenic factor, which is involved in stimulation, proliferation and differentiation of endothelial cells and vascular smooth muscle cells (Benoit et al. 1999; Wagner 2011; Egginton 2009).

Angiogenesis through sprouting is realised by an increase in matrix metalloproteinases (MMPs), required for remodelling of the extracellular matrix, and elevated VEGF levels (Olfert et al. 2016). Indeed, acute resistance exercise induces increases in VEGF and VEGF receptor 2 (VEGFR2/Flk-1) (Gavin et al. 2007). VEGF is also elevated in 2-week-overloaded rat muscles (Degens et al. 2003; Rivilis et al. 2002) to return to baseline levels at 28 days after induction of overload (Rivilis et al. 2002). While eNOS is not thought to play a role in rat models of overload-induced angiogenesis (Olfert et al. 2016), the resistance-exercise-induced angiogenesis in humans was associated with elevated eNOS and hypoxia inducible factor-α (HIF1-α) protein levels (Holloway et al. 2018). The discrepancy between the animal models and the human response may be related to elevated blood flow during resistance exercise that is likely more pronounced than the reported threefold increase in resting blood flow in overloaded muscles (Armstrong et al. 1986).

The significance of VEGF for both overload- and shear-stress-induced angiogenesis is further reflected by the observation that VEGF trapping abolished angiogenesis in both conditions (Williams et al. 2006). Myofibre-derived VEGF, the main source of VEGF, is not essential for maintenance of the capillary bed as seen in mice with myofibre-specific conditional VEGF deletion (Knapp et al. 2016). Total VEGF deletion (Tang et al. 2004), however, does lead to capillary rarefaction, indicating that VEGF plays an essential role not only in angiogenesis, but also in maintenance of the capillary bed.

## Special conditions

### Chronic heart failure

The majority of chronic heart failure (CHF) cases are characterised by left ventricular systolic dysfunction and a reduced ejection fraction (Sullivan and Hawthorne 1995). The muscle hypothesis of CHF suggests that much of the exercise intolerance of patients with CHF is related to changes in skeletal muscle, including fibre atrophy, an increased proportion of type II fibres, and a reduced oxidative capacity and C:F (Hirai et al. 2015; Rogers 2001). The cause of these changes in skeletal muscle during CHF are not clear, but are at least partly attributable to disuse. In addition, left ventricular dysfunction is associated with a rise in catabolic factors, such as elevated tumour necrosis factor-α, and insulin resistance that blunts anabolism. Over time these conditions lead to skeletal muscle myopathy and fatigue. A shift to anaerobic metabolism due to disuse and poor oxygenation leads to an earlier onset of metabolite accumulation during exercise that enhances ventilation via the ergoreflex. This in turn leads to excitation of the sympathetic system, which induces vasoconstriction aggravating the reduction in peripheral blood flow due to cardiac dysfunction and elevating the afterload that in turn further worsens left ventricular function (Coats 1996; Rogers 2001). In addition to the reduced peripheral blood blow resulting from cardiac dysfunction and sympathetic-induced vasoconstriction, the increased blood flow to the respiratory muscles due to heightened ventilation, further diminishes the cardiac output diverted to the periphery and hence perfusion of the locomotory muscles, all negatively impacting the exercise tolerance of patients with CHF.

In most CHF patients, the blood flow to the muscle during single-leg knee-extension exercise is not limited by cardiac output, yet it has been suggested that both impaired convective and diffusive oxygen transport impaired the lower oxygen consumption during single leg exercise in CHF patients (Esposito et al. 2010). Although the authors concluded a lower diffusive oxygen transport played a role in their impaired oxygen consumption during single leg exercise, the C:F ratio was similar to that of controls (Esposito et al. 2010), something also seen by others in humans (Niemeijer et al. 2018) and rodents (Degens et al. 2002). Most studies, however, do report a lower density of the capillary network in muscles of patients with CHF (Hirai et al. 2015; Poole et al. 2012) that was related to their lower maximal oxygen uptake (Duscha et al. 1999). Nevertheless, even in the absence of capillary rarefaction, diffusion limitations may be a consequence of reduced RBC flux and velocity, and an increased proportion of capillaries with intermittent RBC flux both at rest (Kindig et al. 1999) and during contractile activity (Richardson et al. 2003) as seen in rats with CHF. This may precipitate a mismatch between oxygen delivery and utilisation both at rest and during exercise (Hirai et al. 2015).

In an experimental rabbit model of heart failure induced by coronary artery ligation it was seen that vascular rarefaction and apoptosis progressed over time (Nusz et al. 2003). In rats with monocrotaline-induced CHF it was found that apoptosis in the endothelium preceded muscle apoptosis (Vescovo et al. 1998) and the authors suggested that loss of capillaries may lead to inadequate nutritional flow to the myofibres, inducing myofibre apoptosis. In addition, to meet the energy demands during exercise in the face of impaired oxygen delivery and diffusion problems due to capillary rarefaction, there is an increased reliance on anaerobic glycolysis and an earlier onset of muscle fatigue (Poole et al. 2012; Coats et al. 1994; Hirai et al. 2015). These observations suggest that the microcirculation is a potential target to improve exercise tolerance in patients with CHF.

Exercise intolerance is a major cause of further inactivity in CHF patients, and improvements to oxygen delivery and diffusion capacity in the muscle likely improve exercise tolerance. Exercise training not only improves oxygen delivery to the muscle, but also the diffusion capacity that could partly be due to an increase in skeletal muscle CD (Hirai et al. 2015). Indeed, it has been observed in patients with CHF that 8 weeks of knee extensor training resulted in an increased mitochondrial volume density, C:F and number of capillaries around a fibre, indicating angiogenesis (Esposito et al. 2011). These training-induced changes led to both improved knee extension and whole-body exercise capacity without an improvement in maximal cardiac output, supporting the muscle hypothesis of exercise intolerance in CHF and the important role of the microcirculation in exercise performance in these patients. These are encouraging observations as they demonstrate that the microcirculation can be improved in CHF patients, for instance through small muscle training, to enhance whole body exercise capacity (Esposito et al. 2011; Hirai et al. 2015).

### Ageing

During ageing, there is a progressive reduction in exercise capacity, which is evident in a reduction in maximal oxygen consumption (VO_2_max). While largely due to a reduction in cardiac output, this decline in VO_2_max is also attributed to age-related muscle atrophy (Degens 1998; Fleg and Lakatta 1988). In addition, there is a significant ageing-related reduction in C:F in type II fibres that showed atrophy, but neither atrophy nor a reduction in C:F in type I fibres (Barnouin et al. 2017). Interestingly, it has been reported that sarcopenic older people had a lower C:F ratio than non-sarcopenic people (Prior et al. 2016) and it has been suggested that capillary rarefaction may contribute to, and even precede, the age-related muscle atrophy and decline in exercise capacity (Prior et al. 2016; Larsson et al. 2019).

In many rodent studies that assess age-related change in the microcirculation no reductions in C:F are found, and in many cases even no atrophy (Faber et al. 2011; Larsson et al. 2019; Ballak et al. 2016). In one study even an increase in C:F was found (Davidson et al. 1999). While this increase may be due to the reduction in fibre number, which would increase C:F without capillary proliferation, current data do not suggest that capillary rarefaction is involved in the onset of sarcopenia in rodents. This conclusion is probably somewhat premature, as most of these studies are plagued by the use of a young control group that is not yet fully matured and an old group that is only in the initial stage of ageing. This is important as rodent muscles continue maturation throughout the first year of life (Maltin et al. 1989) and may only start to show signs of ageing after the age of 22 months (Lushaj et al. 2008), thus making comparisons akin to a 17-year-old with a 60-year-old person, that mask the effects of age. For example, the similar fibre size and C:F in the plantaris muscle of 5- and 25-month-old rats could falsely lead to the conclusion that 25-month-old rats do not yet show signs of ageing. Yet, the fibre size and C:F were lower in the muscles of 25- than 13-month-old rats (Degens et al. 1993, 1994; Larsson et al. 2019). Thus, to study the effects of ageing, it is important to choose a fully matured age as the young control group and animals that show at least early signs of ageing, such as atrophy and loss of force generating capacity, in the old group to make appropriate comparisons with human ageing (Ballak et al. 2014).

In contrast to rodent studies, most human studies report an ageing-related muscle fibre atrophy and reduced C:F, of particularly type II fibres (Larsson et al. 2019; Barnouin et al. 2017). Overall, it appears that the reduction in C:F, indicating capillary rarefaction, is proportional to the decline in fibre size as reflected by the maintained CD (Parizkova et al. 1971; Larsson et al. 2019). The reduction in C:F during ageing may underestimate the real extent of capillary rarefaction if there is also a concomitant ageing-related loss of fibres, which without capillary loss would result in an increased C:F (Larsson et al. 2019). In fact, in a 12-year longitudinal study of sedentary men from age 65 to 77 reductions in C:F and CD were seen, without a decrease in fibre size, but a loss of overall muscle size, indicative indeed of a reduction in fibre number (Frontera et al. 2000). Therefore capillary loss during ageing is probably more significant than indicated by the reduction in C:F (Larsson et al. 2019).

In old rodent muscles the absence of significant ageing-related changes in C:F and CD in the face of reduced oxidative capacity suggests a capillary supply in relative excess to oxidative capacity (Hepple and Vogell 2004). In human muscle, however, the maximal oxygen consumption supported by a capillary did not change with age (Barnouin et al. 2017), and even when a reduction in oxidative capacity was found, the CD was reduced (Coggan et al. 1992). This suggests that the superfluous capillary supply in ageing rodents is not found in humans, or only occurs later during accelerated denervation with incomplete reinnervation (Larsson et al. 2019). This deserves further study, but the excessive capillary supply in relation to the oxidative capacity during the first 4 weeks after denervation in both the soleus (Degens et al. 2008) and gastrocnemius (Paudyal et al. 2018) muscles supports this proposition.

As previously stated, while C:F and CD are useful in determining capillary supply to skeletal muscle, the distribution of the capillaries in the tissue is also crucial for adequate muscle oxygenation (Degens et al. 2006). While the impact of ageing on heterogeneity of capillary spacing has not been studied extensively, it has been reported that old rats exhibited an larger heterogeneity of capillary spacing than young rats, which was related to the larger variation in muscle fibre size (Degens et al. 2009). Also in human muscle a relationship was found between the variation in fibre size and the heterogeneity of capillary spacing in a muscle, but neither the variation in fibre size nor the heterogeneity of capillary spacing differed between muscles from young-adult and older people (Barnouin et al. 2017). This suggests that not only angiogenesis, but also capillary rarefaction is a controlled process to ensure an adequate tissue oxygenation.

The impaired exercise-induced vasodilation and blood flow, even in endurance-trained people (Proctor et al. 1998; Hildebrandt et al. 2017), is to a large extent attributable to endothelial dysfunction. As VEGF expression is increased under conditions of elevated flow and hence higher sheer stress (Olfert et al. 2016; Milkiewicz et al. 2001) an impaired vasodilation may underlie the reduced VEGF expression in old age and the slow but progressive loss of capillaries (Larsson et al. 2019). As discussed above, conditional deletion of VEGF led to capillary rarefaction and apoptosis (Tang et al. 2004), indicating the importance of VEGF for maintenance of the capillary bed and protection against apoptosis. Interestingly, the large majority of apoptotic nuclei in old mouse muscles belonged to endothelial cells and a large number of SCs (Wang et al. 2014). This resembles the situation in experimental heart failure in rodents where apoptosis of endothelial cells preceded muscle atrophy (Vescovo et al. 1998). In addition to lower VEGF expression in muscles of the elderly, endothelial cells have been shown to become desensitised to VEGF due to the loss of NAD+ dependent sirtuin deacylase 1 (SIRT1) activity in mouse endothelial cells (Das et al. 2018). Accordingly, treatment with an NAD+ booster, nicotinamide mononucleotide (NMN), that activated SIRT-1 led to enhanced VEGF sensitivity, stimulated angiogenesis and improved exercise capacity in old mice (Das et al. 2018). Dietary restriction also leads to angiogenesis via SIRT1 activation (Das et al. 2018; Longchamp et al. 2018). Such changes and the reduction in the VEGF receptor Flk-1 may underlie the attenuated angiogenic response and hypertrophy in overloaded muscles from old mice (Ballak et al. 2016).

In addition to an attenuated exercise-induced vasodilation also the vasodilatory response to insulin is diminished (Fry and Rasmussen 2011). The endothelial dysfunction and capillary rarefaction may contribute to the ageing-related decline in muscle size and strength as it may hamper the delivery of amino acids to the muscle (Timmerman et al. 2010; Groen et al. 2014; Fry and Rasmussen 2011) and contribute to the anabolic resistance in old age (Rennie 2009).

In addition to a reduction in SC content in type II fibres, the distance between type II fibre associated SCs and capillaries is greater in the muscle of older than in those from young men (Verdijk et al. 2007; Nederveen et al. 2016). This greater distance between capillaries and SCs is associated with an impaired SC function in old age (Snijders et al. 2014). Evidence for the importance of the proximity of capillaries to SCs comes from the observation that SC activation after damaging exercise is better when capillaries and SCs are in close proximity (Nederveen et al. 2018). Thus, the increased distance between SCs and capillaries in ageing (Nederveen et al. 2016) could be instrumental in impaired muscle health. Adequate type II fibre capillarisation was found to be crucial in increasing SC content and muscle fibre size in elderly men subjected to resistance training (Snijders et al. 2017a). A recent study by Snijders et al. (2019) found that adhering to a 12-week program of exercise training in healthy older men led to an increase in skeletal muscle capillarisation, which correlated with increased SC content at 24 h post exercise. This shows that prolonged exercise training can increase the number of capillaries per type II fibre, which is associated with improved SC and hypertrophic responses, providing further evidence for the role of capillaries in hypertrophy.

Part of the decrement in muscle mass and function is attributable to disuse (Degens and Alway 2006). Master athletes maintain high levels of physical activity (Hannam et al. 2017) and are a useful model to disentangle the effects of disuse from those of ageing per se (Rittweger et al. 2004). It appears that regular physical activity can largely attenuate or even prevent ageing-related changes in skeletal muscle microcirculation. For instance, the C:F was higher, fibre size larger and oxidative capacity similar in activity-matched older than young endurance athletes (Coggan et al. 1990), and in master cyclists there was no significant ageing-related reduction in fibre size and C:F (Pollock et al. 2018). Even in previously sedentary older people endurance and strength training can induce angiogenesis (Hepple et al. 1997; Holloway et al. 2018; Snijders et al. 2019). These observations indicate that indeed a large part of the decrement in the density of the capillary network is attributable to disuse. One thing to consider, however, is that the hypertrophy and SC activation in response to resistance exercise has been reported to be less in older people with a lower CD (Snijders et al. 2017a). The implication is that benefits of resistance exercise in older people may be helped if preceded or performed concurrently with endurance exercise, which is a potent stimulator of angiogenesis inducing also an increase in CD (Hepple et al. 1997).

## Concluding remarks

Not only the quantity, but also the distribution of capillaries is important for adequate muscle oxygenation. Interestingly, the main determinant of capillary supply to a fibre is not the oxidative capacity, but rather the size of the fibre. Indeed, muscle hypertrophy and angiogenesis follow a similar time course, and the attenuated hypertrophy in old age is at least partly attributable to a diminished density of the capillary network and impaired angiogenesis. Endothelial dysfunction and capillary rarefaction most likely contribute to the exercise intolerance, may precede muscle atrophy and a greater reliance on glycolytic metabolism during CHF and ageing. Therefore, we suggest that the microcirculation is an important target for rehabilitation in CHF and combating sarcopenia.
